# Comparative Analysis of Gut Microbiota Between Fast-Growing and Slow-Growing Short-Finned Eels, *Anguilla bicolor pacifica*, and the Application of *Bacillus tropicus* FG2 as a Probiotic to Enhance Growth Performance of Eels

**DOI:** 10.3390/ani16010054

**Published:** 2025-12-24

**Authors:** Yi-Yuan Liang, Shao-Yang Hu, Chun-Hung Liu

**Affiliations:** 1 Department of Aquaculture, National Pingtung University of Science and Technology, Pingtung 912301, Taiwan; iyuan.liang.iyl@gmail.com; 2 Department of Biological Science and Technology, National Pingtung University of Science and Technology, Pingtung 912301, Taiwan; syhu@mail.npust.edu.tw

**Keywords:** *Anguilla bicolor pacifica*, gut microbiota, probiotic, *Bacillus tropicus*, growth performance

## Abstract

This study aims to investigate the differences in microbial community structure between fast-growing (FG) and slow-growing (SG) short-finned eels, *Anguilla bicolor pacifica*, using high-throughput 16S rDNA sequencing, and to evaluate the potential probiotic properties of *Bacillus tropicus* isolated from eel intestinal microbiota to enhance growth performance. High-throughput 16S rDNA sequencing revealed no significant differences in the α-diversity between FG and SG eels. Bacterial genera such as *Cetobacterium*, *Clostridium*, and *Bacteroides* were predominant in both groups, with *Edwardsiella*, *Aeromonas*, and *Fusobacterium* being more abundant in SG eels, suggesting a higher presence of potential pathogens. The analysis of the relative abundance of gut microorganisms revealed that SG eels harbored higher levels of potentially pathogenic bacteria, including *Edwardsiella tarda* and *Aeromonas jandaei*. In contrast, FG eels exhibited a greater abundance of the potential probiotic *B. tropicus*. Six strains of bacteria with relative abundance were isolated from the FG group, displaying superior digestive enzyme activity, including protease, lipase, amylase, cellulase, xylanase, and phytase, particularly strain FG2. Phylogenetic analysis confirmed that FG2 was closely related to *B. tropicus*. A virulence assessment confirmed the non-pathogenic nature of *B. tropicus* FG2, supporting its probiotic potential. Furthermore, feeding eels a diet supplemented with *B. tropicus* FG2 significantly enhanced growth performance, as evidenced by increased final weight percentages of weight gain and total production per tank (*p* < 0.05), while the proximate composition of the dorsal muscle showed an increase in lipid content (*p* < 0.05). These findings highlight *B. tropicus* FG2 as a promising probiotic for aquaculture applications.

## Simple Summary

The gut microbiome strongly affects animal growth, physiology, and overall health. A balanced and diverse intestinal microbial community improves feed digestion, nutrient absorption, and gut barrier function, making microbiome stability a key factor in aquaculture performance. This study compares the intestinal microbiota of fast- and slow-growing eels to identify bacterial taxa linked to superior growth. Certain gut commensals may promote growth by enhancing digestion, producing enzymes, or regulating metabolic compounds. By isolating these beneficial strains, we aim to develop physiologically based probiotics for eel culture. The findings provide microbiome insights and functional strains that support evidence-based probiotic applications, offering practical value for improving eel health and farming efficiency.

## 1. Introduction

Eels are highly esteemed in aquaculture owing to their significant market value, robust survival rates, high productivity, and extensively researched disease profiles. Among the 19 recognized species of *Anguilla*, the Japanese eel, *Anguilla japonica*, and the European eel, *A. anguilla*, hold the greatest economic and cultural significance, being primarily cultivated in East Asia (notably Japan, Taiwan, and China) and Europe (particularly Italy and the Netherlands), respectively [1]. Despite their significance, the juvenile populations of *A. anguilla* and *A. japonica* have declined dramatically in recent decades, as reported by Pike et al. [2] and Hakoyama et al. [3,4]. Consequently, the urgent need to develop techniques for the large-scale production of eel larvae or to identify alternative *Anguilla* species for *A. japonica* aquaculture has become increasingly apparent.

The short-finned eel, *A. bicolor*, is a tropical catadromous species of considerable economic significance in Southeast Asian fisheries. Recognized as a viable alternative to *A. japonica* for aquaculture, *A. bicolor* is highly valued for its texture and flavor, which align with market preferences and enhance its commercial appeal [5]. Beyond its culinary desirability, *A. bicolor* exhibits a broader geographic distribution than most of the 19 recognized species and subspecies within the genus *Anguilla* [6,7]. Currently, two subspecies of *A. bicolor* are distinguished: *A. bicolor bicolor* and *A. bicolor pacifica* [6,8,9]. While these subspecies share similar morphological traits, their geographical distributions are distinct. *A. bicolor bicolor* is restricted to the Indian Ocean, whereas *A. bicolor pacifica* is distributed throughout the Indo-Pacific, encompassing the Western Pacific Ocean and the surrounding waters of northern Indonesia, China, Vietnam, the Philippines, Borneo, Sulawesi, and New Guinea [6,7,8]. Notably, *A. bicolor pacifica* is characterized by a relatively stable effective population size [10] and therefore may represent a promising candidate species to help mitigate the current shortage of *A. japonica* glass eel resources for aquaculture. This extensive distribution highlights the ecological and commercial significance of *A. bicolor pacifica* across diverse aquatic ecosystems.

In eel aquaculture, the rearing phase commences when glass eels are collected from estuarine regions. A notable challenge during this phase is the prevalent size heterogeneity among eels [11,12], which can significantly impact the efficiency of farming operations. This variability necessitates substantial labor investment in size-grading procedures to minimize disparities [13,14]. Size heterogeneity often leads to a phenomenon known as the hierarchical size effect, wherein dominant individuals monopolize access to feed resources, inhibiting the feeding opportunities of smaller conspecifics. As a result, larger eels are typically able to consume more feed, exhibit accelerated growth rates, and ultimately achieve greater body weights compared to their smaller counterparts [15].

Size variation among aquaculture animals is influenced by a multitude of factors, including water quality, stocking density, territorial behavior, and nutritional availability [16,17]. These factors interact in intricate ways, shaping growth rates, feed conversion efficiency, and overall health, ultimately resulting in size heterogeneity within a population. Nevertheless, even under identical rearing conditions, growth performance can be further modulated by intrinsic factors, such as the central nervous system, and endocrinological and neuroendocrinological regulatory pathways [18]. These internal mechanisms play pivotal roles in mediating physiological responses to environmental cues, thereby contributing to individual variability in growth outcomes. Intestinal microbiota is known a new possible internal factor as it is gradually recognized that it plays very important role in the health and growth of the host [19,20]. Sun et al. [21] conducted a comprehensive investigation into the gut microbiota differences between slow-growing (SG) and fast-growing (FG) juvenile *Epinephelus coioides*. Their findings revealed a higher abundance of *Vibrio* species in SG groupers, whereas the gut microbiota of FG groupers was predominantly enriched with beneficial bacterial species, including *Bacillus pumilus*, *B. clausii*, and *Psychrobacter* sp., which were successfully isolated and identified. Building on these findings, Sun et al. [22] demonstrated that *B. pumilus* and *B. clausii* could be effectively incorporated into grouper diets as probiotics, leading to improved growth performance and enhanced health status. Shi et al. [23] also explored the differences in gut microbiota of European eel, *A. anguilla* and identified *Cetobacterium* as the predominant genus in the intestinal microbiota of fast-growing individuals. This bacterial genus was suggested as a potential probiotic for European eels due to its association with enhanced growth performance. However, despite this promising observation, no follow-up studies have been conducted to validate the probiotic application of *Cetobacterium* in European eel aquaculture. Further advancing this field, Zhang et al. [12] investigated the gut microbiota of American eel, *A. rostrata* and observed a higher relative abundance of Firmicutes, a phylum known for producing short-chain fatty acids (SCFAs), in FG individuals compared to their SG counterparts. This finding suggests that the gut microbiota in FG eels is more efficient in fermenting non-digestible polysaccharides, thereby generating higher levels of SCFAs, which serve as critical energy sources for the host. Despite these promising results, Zhang et al. [12] did not isolate specific bacterial strains from eel intestines to directly evaluate their probiotic potential. Collectively, these studies underscore the pivotal role of gut microbiota in modulating growth performance in aquaculture species. However, further research is needed to isolate and characterize candidate probiotic strains, assess their functional roles, and validate their efficacy in controlled aquaculture settings.

This study aims to comprehensively investigate the differences in gut microbiota between short-finned eels with varying growth rates. Specifically, it seeks to isolate and identify distinct bacterial strains with potential probiotic properties and assess their efficacy in promoting growth performance. Ultimately, this research aspires to mitigate growth rate disparities and size heterogeneity, addressing key challenges in eel aquaculture and contributing to more sustainable and efficient production practices.

## 2. Materials and Methods

### 2.1. Experimental Fish and Acclimation

Short-finned eels, *A. bicolor pacifica*, were obtained from a private eel farm in Pingtung, Taiwan. To investigate differences in gut microbiota between FG and SG eels, eels reared under identical conditions for 8 months in the same tank were immediately used for DNA extraction and probiotic isolation upon arrival at the laboratory. For the evaluation of probiotic efficacy on growth performance, juvenile eels (initial weight: 10.2 ± 0.5 g) were acclimated in a 4-ton tank (2 m × 2 m × 0.8 m; L × W × H) with a continuous water flow rate of 2.5 L min^−1^ for two weeks prior to the experiment. The water depth was maintained at 0.3 m, corresponding to an effective water volume of approximately 1.2 tons. During this acclimation period, the eels were fed a commercial dough diet twice daily at ~5% of their body weight.

### 2.2. Gut Microbiota Between FG and SG Eels

Eels weighing less than 100 g were classified as SG (3 fish), while those exceeding 500 g were classified as FG (3 fish). Before dissection, eels were anesthetized in ice water until they showed no movement. The intestines of SG (48.3 ± 8.5 g) and LG (633.5 ± 34.4 g) eels were carefully dissected on ice to preserve sample integrity. The intestinal tracts were meticulously emptied of luminal contents through gentle manual pressure, followed by three sequential rinses with sterile saline solution to ensure complete removal of residual material. A sterilized pair of fine surgical scissors was employed to excise the middle intestinal segment, which was subsequently placed on a sterile Petri dish. Remaining fecal residues were carefully extracted using sterile disposable pipettes and spoons to prevent cross-contamination. Intestinal mucus was then meticulously collected by scraping the inner surface of the intestinal tract with sterile instruments and immediately transferred into 1.5 mL tubes. To preserve sample integrity and prevent DNA degradation, the mucus samples were promptly processed for DNA extraction. A total of nine eels from each group were used in this analysis. For each biological replicate, mucus samples from three individual eels were pooled, resulting in three composite samples (three pools × three eels per pool). DNA extraction for each of the three pooled samples was performed using the DNeasy^®^ PowerSoil^®^ Pro Kit (Qiagen, Hilden, NRW, Germany) in strict accordance with the manufacturer’s protocol. DNA purity and concentration were evaluated using a NanoDrop spectrophotometer (MN-193, Maestro Gen Inc., Hsinchu, Taiwan), with the 260/280 absorbance ratios ranging from 1.86 to 1.95 and the 260/230 ratios ranging from 2.07 to 2.17, indicating high purity and minimal contamination. Sample quality and integrity were further verified using the Agilent 4200 TapeStation system (Agilent Technologies, Palo Alto, CA, USA), which yielded DNA Integrity Number (DIN) values of 9.2–9.6.

The full-length 16S rRNA gene was amplified from microbial genomic DNA extracted from samples using the universal primer pair 27F (AGRGTTYGATYMTGGCTCAG) and 1492R (RGYTACCTTGTTACGACTT), along with the KAPA HiFi HotStart Ready Mix PCR kit (KAPA Biosystems, Wilmington, MA, USA). The resulting PCR amplicons were evaluated for size distribution and concentration using the D5000 ScreenTape Assay on an Agilent TapeStation 4200 system (Agilent Technologies, USA). Subsequently, the pooled PCR products were purified with SMRTbell^®^ cleanup beads (PacBio Biosciences, Menlo Park, CA, USA) and prepared into barcoded single-molecule real-time (SMRT) libraries using the SMRTbell^®^ prep kit 3.0 (PacBio Biosciences, Menlo Park, CA, USA). The libraries underwent an additional purification step with SMRTbell^®^ cleanup beads, followed by quantification using the Qubit 1X dsDNA High Sensitivity Kit (Thermo Fisher Scientific, Waltham, Mass, USA). Their size distribution was further verified with the D5000 ScreenTape Assay. Sequencing was performed on the PacBio Sequel IIe platform (PacBio Biosciences, Menlo Park, CA, USA), and the raw sequencing reads were processed into high-quality circular consensus sequences (CCS) using the CCS module within SMRT Link software version 25.3 (PacBio Biosciences, Menlo Park, CA, USA).

Taxonomic classification was achieved using the RDP Naive Bayesian Classifier algorithm [24] with a minimum bootstrap confidence threshold of 50. The reference dataset included NCBI RefSeq records under BioProject 33175 and 33317 [25], supplemented by the Ribosomal Database Project (RDP) [26]. Prior to downstream analyses, low-abundance amplicon sequence variants (ASVs) were filtered out by removing ASVs with fewer than 20 total reads across all samples. This filtering step was implemented to eliminate potential sequencing artifacts and spurious rare variants that may compromise diversity estimation and ordination accuracy. Principal coordinates analysis (PCoA) was performed using the Bray–Curtis dissimilarity matrix on the Metware Cloud platform (Metware Biotechnology Co., Ltd., Wuhan, China). Key diversity indices, including Shannon’s diversity index, and Margalef’s species richness (d), were calculated using QIIME (http://qiime.org/scripts/alpha_diversity.html (accessed on 8 December 2024)). Patterns of microbial dominance and cumulative species contributions were analyzed with the Plymouth Routines in Multivariate Ecological Research (PRIMER) software (Version 6.1.5).

### 2.3. Potential Probiotic Isolation from the Intestine of FG Eel

The intestinal mucus used for DNA extraction was also employed for the isolation of potential probiotics using nutrient agar (NA) (Difco TM Nutrient Agar, Spark, MD, USA). Briefly, the intestinal mucus was mixed with sterile saline solution (0.85% NaCl) and subsequently spread on NA following serial dilutions. The plates were then incubated at 27 °C for 4 days. Differences in colony morphology and colony count between FG and SG eels were observed. Distinct or abundant colonies were selected and transferred to fresh NA plates for further analysis.

A total of 10 colonies (designated as FG1 to FG8 (isolated from FG eels), and SG9 to SG10 (isolates from SG eels)) exhibiting differences between FG and SG groups, were sent to Tri-Biotech, Inc. (Taipei, Taiwan) for species identification. Identification was performed using 16S rDNA and gyrase B (*gyrB*) gene sequencing with the following primers: forward, 5′-GTGCCAGCAGCCGCGGTAA-3′, and reverse, 5′-GACTACCAGGGTATCTAATC-3′, and forward, 5′-ACATCGTGAAGGTAAAATCC-3′, and reverse, 5′-TCTCCGCCAATGTCGTACAT-3′, respectively. The obtained 16S rDNA and *gyrB* sequences were compared against known sequences in the GenBank database using the Basic Local Alignment Search Tool (BLAST) (NCBI Nucleotide BLAST, https://blast.ncbi.nlm.nih.gov/Blast.cgi, accessed on 6 September 2024). Subsequently, a phylogenetic tree was constructed using Molecular Evolutionary Genetics Analysis (MEGA) software version 4.1 (available online: http://www.megasoftware.net/, accessed on 6 September 2024). The tree was generated using the neighbor-joining method, with bootstrap values calculated from 1000 replicates. The tree was drawn to scale, with branch lengths proportional to evolutionary distances.

### 2.4. Selection of Probiotic Candidates Based on Digestive Enzyme Activity

Strains FG1~FG8 were selected for the analyzing digestive enzyme activity, while strains SG9 and SG10 were excluded from further investigation because their identification as potential pathogens, *Aeromonas caviae* and *A. veronii*, respectively. Initially, all selected strains underwent protease activity assessment. Strains exhibiting significantly higher protease activity were subsequently subjected to additional enzymetic assays to further characterize their digestive enzyme profiles. The strain exhibiting superior digestive enzyme activity was identified as a potential probiotic candidate and was subsequently evaluated for its probiotic efficacy in enhancing eel growth performance.

Prior to the assay of enzymatic activity, tested strains were inoculated in nutrient broth (NB) and incubated for 24 h at 150 rpm at 27 °C. Thereafter, bacteria were centrifuged for 20 min at 2840× *g* at 4 °C. The supernatant was transferred to a new tube and used for analysis of enzymatic activity.

#### 2.4.1. Assessment of Enzymatic Activity

The protease activity assay was performed following the methodology described by Beg et al. [27] with slight modifications. In brief, protease activity was measured in triplicate at 27 °C using a 100 mM Tris-HCl buffer (pH 9.0). Sample (100 μL) was incubated with 100 mL of a 1% (*w*/*v*) casein solution prepared in Tris-HCl buffer (pH 9.0) for 10 min at 27 °C. The enzymatic reaction was terminated by adding 500 mL of 5% (*v*/*v*) trichloroacetic acid (TCA). The mixture was then centrifuged at 3000× *g* for 20 min, and the products were determined using a modified Lowry’s method. One unit of protease activity was defined as the amount of enzyme required to liberate 1 μg of tyrosine mL^−1^ min^−1^ under the specified assay conditions.

Lipase activity was assessed using the titrimetric method described by Teitz and Friedreck [28], with minor modifications. Briefly, 1.5 mL of a stabilized olive oil emulsion, serving as the lipase substrate, was evenly mixed with 1.5 mL of 0.1 M Tris-HCl buffer (pH 8.0). Subsequently, 1 mL of sample was added to the mixture, followed by incubation at 27 °C for 6 h. The enzymatic reaction was terminated by adding 3 mL of 95% ethanol. Released free fatty acids were then titrated using 0.01 N NaOH with 0.9% (*w*/*v*) thymolphthalein in ethanol as an indicator. A blank control was prepared following the same protocol, except that the crude enzyme extract was added immediately prior to titration to account for background corrections. One unit of lipase activity was defined as the amount of 0.05 N NaOH required to neutralize the fatty acids liberated from the substrate during the incubation period, after correction for the blank.

Amylase activity was evaluated using the dinitrosalicylic acid (DNS) method, as described by Soy et al. [29], with slight modifications. Briefly, 500 μL of sample was combined with 1 mL of 0.01 M sodium phosphate buffer (pH 6.9) containing 0.5% soluble starch as the substrate. The reaction mixture was incubated at 25 °C for 10 min to allow enzymatic hydrolysis. After incubation, reducing sugars were quantified using the DNS reagent, and the absorbance of the resulting solution was measured spectrophotometrically at 540 nm. One unit of amylase activity was defined as the amount of enzyme required to liberate 1 μmol of maltose per min under the assay conditions (pH 6.9, 25 °C).

Cellulase activity was quantified by measuring the release of reducing sugars, expressed as glucose equivalents, using the DNS method, as described by Gascoigne and Gascoigne [30], with slight modifications. Briefly, 45 μL of 1% carboxymethyl cellulose prepared in sodium citrate buffer (pH 6.0) was combined with 10 μL of enzyme sample. The reaction mixture was incubated at 55 °C for 15 min to facilitate enzymatic hydrolysis. Upon completion of the incubation, 50 μL of DNS reagent was immediately added, and the mixture was then heated in a boiling water bath for 5 min to allow the formation of a chromogenic complex, followed by cooling to room temperature. The absorbance of the resulting solution was measured at 540 nm using a spectrophotometer (V-630, Jasco, Jasco Corporation, Tokyo, Japan). One unit of cellulase activity was defined as the amount of enzyme required to release 1 μmol of glucose per min under the specified assay conditions.

Xylanase activity performed following the method described by Bailey et al. [31], with slight modifications. Briefly, the reaction mixture comprised 1.8 mL of a 1.0% (*w*/*v*) suspension of beechwood xylan prepared in 50 mM sodium citrate buffer (pH 6.0) and 0.2 mL of enzyme sample in 50 mM sodium citrate buffer (pH 6.0). The mixture was incubated at 60 °C for 5 min to facilitate enzymatic hydrolysis. Subsequently, 3 mL of DNS reagent was added to terminate the reaction and quantify the released reducing sugars. The mixture was then heated at 99 °C for 15 min, and the absorbance was measured spectrophotometrically at 540 nm. The concentration of reducing sugars was calculated using xylose as the standard. One unit of xylanase activity was defined as the amount of enzyme required to release 1 μmol of reducing sugar, expressed as xylose equivalents, per min under the assay conditions.

Phytase activity was assessed by mixing 500 µL of the sample with 250 µL of 0.2 M sodium acetate buffer (pH 5.15) and 5 mM sodium phytate. The reaction mixtures were incubated at 27 °C in a water bath for 45 min to facilitate enzymatic activity. To terminate the reaction, 1 mL of 15% (*w*/*v*) TCA was promptly added. Subsequently, 500 µL of the reaction mixture was mixed with 4 mL of freshly prepared colorimetric reagent, consisting of acetone, 10 mM ammonium molybdate, and 5 N sulfuric acid in a 2:1:1 (*v*:*v*:*v*) ratio, along with 400 µL of 1 M citric acid. The released inorganic phosphate was quantified spectrophotometrically at a wavelength of 355 nm. A standard curve was constructed using potassium dihydrogen phosphate (KH_2_PO_4_) as a standard, following the methodology described by Heinonen and Lahti [32]. One unit of phytase activity was defined as the amount of enzyme required to liberate 1 µmol of inorganic phosphate per min from a 5 mM sodium phytate solution under the assay conditions.

Specific activity for all determined enzyme was expressed as U mg^−1^ of protein, and protein concentration in the sample was determined using a Bio-Rad protein assay kit (no. 500-0006, Bio-Rad Laboratories, Berkeley, CA, USA) with bovine serum albumin (Sigma, Louis, MO, USA) as a standard.

#### 2.4.2. Virulence Assessment for Aquatic Animals

Several aquatic species, including 3 fish and 3 crustacean species (Table 1), were used to assess the virulence of the selected strain FG2 through injection. Test animals were procured from private aquaculture farms, transferred to the aquafarm facility at NPUST, Taiwan, and acclimated for seven days. During the acclimation period, fish and crustacean were fed a commercial diet twice daily.

The assessment was performed in aquaria containing 20 L of water, with each species evaluated in triplicate, and each replicate consisting of 10 animals. Virulence assessment was conducted via injection at a dose of 10^9^ cfu (g weight)^−1^ over a 14-day period. Throughout the experiment, water quality was maintained by daily water renewal. Mortality rates were monitored twice daily, at 9:00 a.m. and 4:00 p.m. Water temperature was maintained at 28 °C using submersible heaters, while dissolved oxygen (DO) levels were kept at ≥5 mg L^−1^ through continuous aeration.

### 2.5. Assessment of Probiotic Efficiency

To evaluate whether the isolated strain FG2 could enhance the growth performance of eels, the bacterium was incorporated into the diet at a level of 10^9^ cfu (kg diet)^−1^, and a 56-day feeding trial was subsequently conducted.

#### 2.5.1. Probiotic Preparation

The selected strains FG2 was inoculated in NA and incubated for 24 h at 27 °C, one colony was then transferred to NB and incubated for 24 h at 150 rpm at 27 °C. Thereafter, bacteria were centrifuged for 20 min at 2840× *g* at 4 °C. The supernatant was discarded and the pellet was washed twice with sterile 0.9% NaCl. The pellets were dissolved in 10% skim milk and freeze-dried (Freeze dryer 5030/8530, Pannchum, Kaohsiung, Taiwan). The probiotic powder was stored at −20 °C until use. The viability of bacteria was determined by the spread plating on NA.

#### 2.5.2. Experimental Diet Preparation

The composition of the experimental diets is presented in Table 2. The protein and lipid contents were formulated to approximate 48% and 10%, respectively, ensuring optimal nutritional balance for the target species. Initially, all feed ingredients were finely ground into powder, sieved through a #60-mesh screen, and homogeneously mixed with fish oil to achieve uniform distribution. The resulting feed mixture was subsequently stored at 4 °C to preserve its integrity until use. Prior to feeding trials, the proximate composition of the diets was analyzed following the standard protocols outlined by the Association of Official Analytical Chemists [33]. Crude protein was determined using the Kjeldahl method with a Kjeltec System (Tecator, Höganäs, Sweden). Crude lipid was measured using the Soxhlet extraction method. Moisture content was analyzed using a moisture analyzer (MX-50, A&D Company, Tokyo, Japan). Ash content was determined by incineration in a muffle furnace at 550 °C. Furthermore, the concentration of probiotics in the diets was quantified using the spread plate method, with bacterial colonies identified. The concentration of *Bacillus* spp. in the control and experimental diets was determined to be 1 × 10^2^ cfu (kg diet)^−1^ and 1.2 × 10^9^ cfu (kg diet)^−1^, respectively, ensuring precise bacterial supplementation in accordance with experimental design criteria.

#### 2.5.3. Fish Rearing

The growth performance trial was conducted in indoor cement tanks with dimensions of 2 m × 2 m × 0.8 m, filled with water to a depth of 0.3 m. Aeration was continuously supplied via air stones, and water flow was maintained at a rate of 0.5 L min^−1^ to ensure DO levels remained above 5 mg L^−1^ and to uphold optimal water quality throughout the experimental period.

Two experimental groups were conducted: a control group without probiotic supplementation and a treatment group supplemented with probiotics at a concentration of 10^9^ cfu (g diet)^−1^. Each group was conducted in triplicate, with 100 juvenile eels allocated per replicate tank. The growth trial was performed for 56 days, during which fish were fed a feed dough prepared by mixing the powdered diet with water at a ratio of 1:1.2 (*w*/*w*) to form a cohesive dough. Feeding was performed twice daily at a feeding rate equivalent to 5% of the body weight. After 2 h of feeding, uneaten feed was carefully collected and dried in an oven at 100 °C. The total feed intake was then calculated by subtracting the weight of the uneaten feed from the total feed provided. Throughout the experiment, 10 fish from each tank were randomly sampled and weighed biweekly to adjust the feeding rate according to their updated body weight. At the end of the feeding trial, individual fish weight and body length were meticulously measured, and growth performance parameters were calculated as follows:

Survival (%) = [(initial number of fish − number of survival fish)/initial number of fish] × 100;

Percentage weight gain (%) = [(final weight − initial weight)/initial weight] × 100;

Feed efficiency = (final weight − initial weight)/total feed provided;

Condition factor = (fish weight/fish length^3^) × 100

#### 2.5.4. Body Composition

Following last weight and length measurements, 6 eels from each group were euthanized by immersion in ice water. The dorsal muscle tissue was carefully dissected, dried in a hot air oven at 70 °C for 24 h, and subsequently stored in a refrigerator for further chemical analysis. Proximate composition, including crude protein, crude lipid, dry matter, and ash content, was determined according to the standard methods outlined by the Association of Official Analytical Chemists [33].

### 2.6. Statistical Analysis

A Completely Randomized Design was used in this study, and juvenile eels were randomly allocated to the experimental tanks corresponding to each dietary treatment. Statistical analyses were performed using SPSS (IBM SPSS Statistics for Windows, Version 22.0, IBM Corp., Armonk, NY, USA). Prior to conducting ANOVA, Levene’s test and the Shapiro–Wilk test were applied to assess homogeneity of variances and normality, respectively. Probiotic enzyme activities were analyzed using one-way ANOVA to evaluate differences among treatments. When significant effects were detected, Tukey’s HSD post hoc test was used to determine pairwise differences among groups. Independent-samples t-tests were used to compare the relative abundance and alpha-diversity indices of gut microbiota between the FG and SG groups, as well as growth performance and the proximate composition of dorsal muscle between the control and probiotic treatment groups. Statistical significance was set at *p* < 0.05.

## 3. Results

### 3.1. NGS of 16S rDNA Gene Libraries and Taxonomic Identity of Microbial Community

Across all samples, a total of 3743 ASVs, comprising 832,857 denoised sequences, were identified in the intestinal microbiota of eels. Statistical analysis of the α-diversity indices, which evaluate species richness and evenness, is summarized in Table 3. The results indicated no statistically significant differences in α-diversity indices between FG and SG eels.

**Figure 1:**
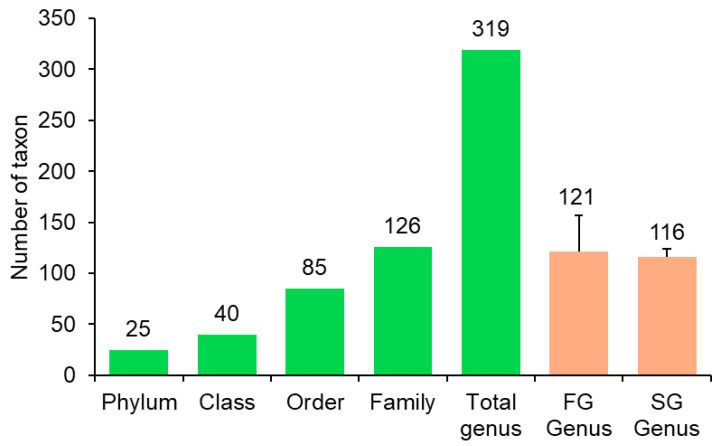
Microbial taxonomic identification in the gut of the FG and SG eels.

**Figure 2:**
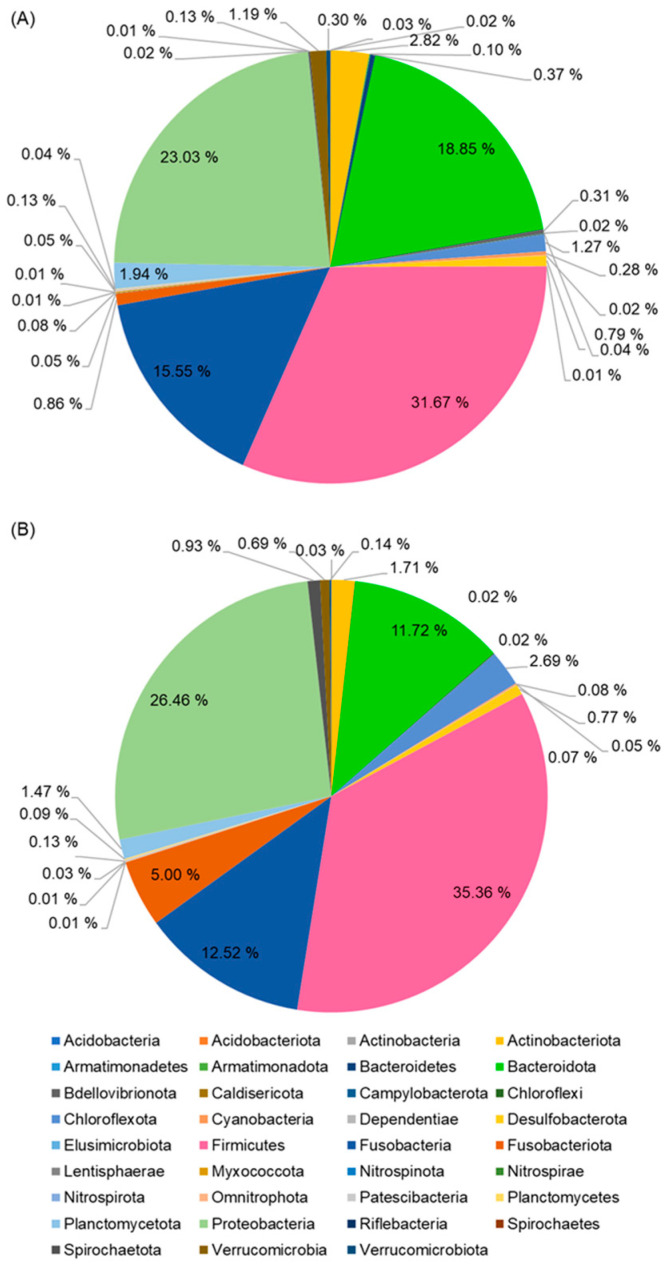
Average phylum distributions of intestinal microbiota of the FG (**A**) and SG (**B**) eels.

As depicted in Figure 1, a total of 319 bacterial genera were identified across intestinal samples from FG and SG eels. Among these, 121 genera were identified in FG eels and 116 genera in SG eels. The bacterial genera identified in both groups predominantly belonged to the phyla Firmicutes and Proteobacteria, with Bacteroidota and Fusobacteria representing minor phyla (Figure 2).

**Figure 3:**
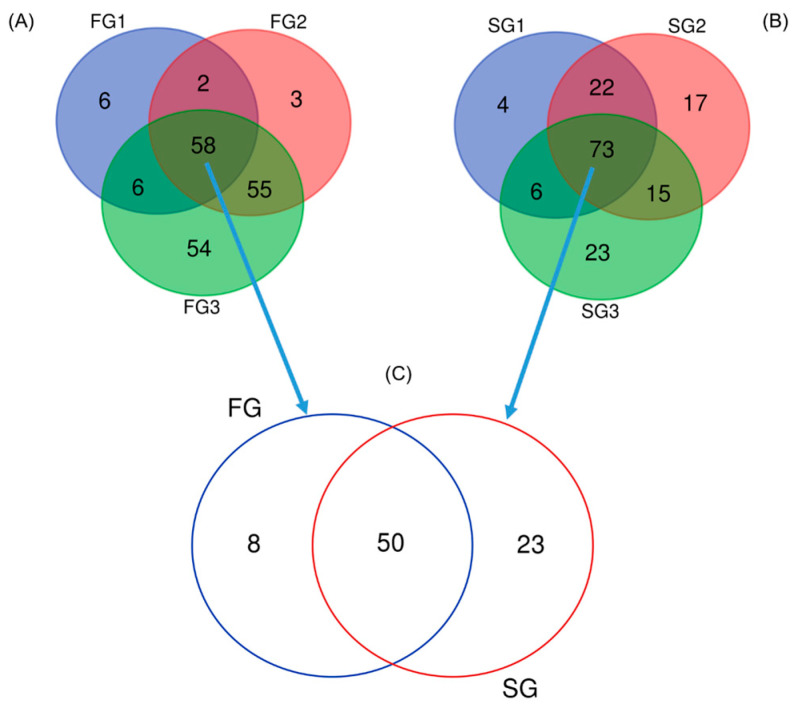
Venn diagrams illustrating the core microbiota composition within replicates of each group (**A**,**B**), and between the FG and SG eels (**C**). The diagrams highlight the shared and unique microbial taxa across replicates and between the two groups, providing insights into the core microbial community in the eel gut.

Figure 3 further illustrates the similarities and differences in bacterial genera observed between the FG and SG groups. Shared ASVs within FG replicates (Figure 3A) and SG replicates (Figure 3B) were 58 and 73, respectively. A total of 50 ASVs were shared as core microbiota between the FG and SG groups (Figure 3C). Taxonomic classifications of unique ASVs at the genus level are shown in Figure 3C. The FG group was characterized by the presence of unique genera such as *Aminipila*, *Tabrizicola*, *Rhodoluna*, *Pseudomonas*, *Acinetobacter*, *Rhodoplanes*, *Shigella*, and *Microcystis*. Conversely, unique genera in the SG group included *Singulisphaera*, *Haloimpatiens*, *Flavobacterium*, *Novosphingobium*, *Reyranella*, *Terrimicrobium*, *Roseomonas*, *Akkermansia*, *Rhodoligotrophos*, *Fictibacillus*, *Sulfurisoma*, *Aureliella*, *Ruthenibacterium*, *Butyricicoccus*, *Bacteriovorax*, *Bellilinea*, *Parabacteroides*, *Mesobacillus*, *Escherichia*, *Lactococcus*, *Croceimicrobium*, *Rhabdaerophilum*, and *Citrobacter*.

**Figure 4:**
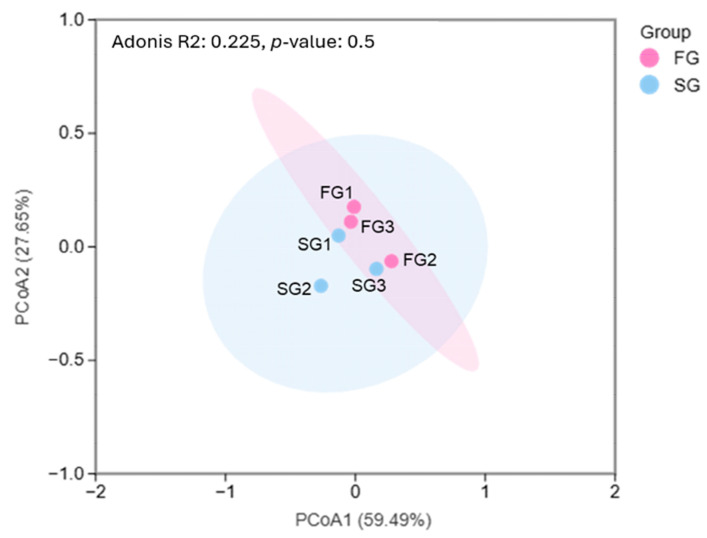
Principal coordinates analysis (PCoA) of gut microbiota at the genus level in FG and SG eels. The PCoA was generated using the Bray–Curtis dissimilarity matrix based on the default workflow of the Metware Cloud platform. Each point represents the gut microbial community of an individual eel, and the shaded ellipses denote the 95% confidence intervals for each group. The clustering patterns reveal differences in gut microbial community structure between FG and SG eels.

To evaluate the similarity of gut bacterial communities, a PCoA based on the Bray–Curtis distance at the genus level was performed. The FG and SG groups exhibited substantial overlap, indicating that the overall gut microbial composition was largely comparable between FG and SG eels. Although minor within-group dispersion was observed, particularly in SG1 and SG2, no distinct separation between the two growth groups was detected. PCoA1 and PCoA2 together explained 59.49% and 27.65% of the total variation, respectively (Figure 4).

**Figure 5:**
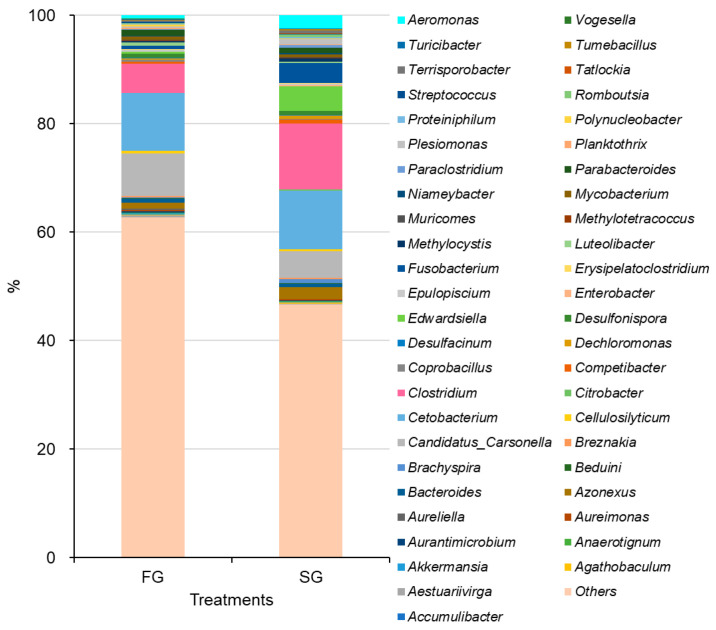
Relative abundances of bacterial genera identified in the intestines of FG and SG eels. Each bar represents the average percentage contribution of the genera within each group. The genera are color-coded, with corresponding labels displayed on the right side of the figure.

At the genus level, the top 10 most abundant bacteria in FG eels were *Cetobacterium*, *Clostridium*, *Bacteroides*, *Parabacteroides*, *Fusobacterium*, *Mycobacterium*, *Aeromonas*, *Candidatus_Carsonella*, *Azonexus*, and *Desulfonispora*. In comparison, the top 10 genera in SG eels included *Clostridium*, *Cetobacterium*, *Parabacteroides*, *Edwardsiella*, *Fusobacterium*, *Aeromonas*, *Plesiomonas*, *Azonexus*, *Candidatus_Carsonella*, and *Desulfonispora* (Figure 5). Among these genera, a higher prevalence of potential pathogens was observed in SG eels. Specifically, *Edwardsiella* (4.49%), *Fusobacterium* (3.67%), *Aeromonas* (2.46%), and *Plesiomonas* (1.02%) were markedly more abundant in SG eels compared to their respective abundances in FG eels (0.39%, 0.64%, 0.57%, and 0.33%, respectively) (Figure 5).

**Figure 6:**
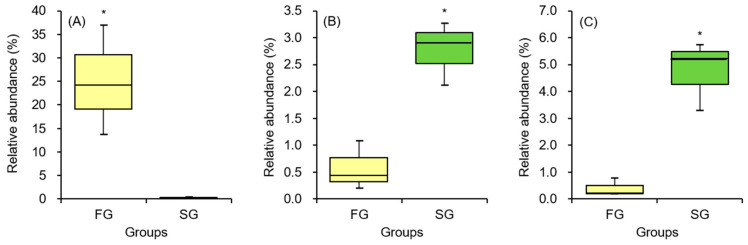
Differentially abundant gut bacterial taxa between FG and SG eels. Relative abundance of *Bacillus tropicus* (**A**), *Aeromonas jandaei* (**B**), and *Edwardsiella tarda* (**C**) in FG and SG eels. Data are presented as box-and-whisker plots, showing the median, interquartile range, and full data distribution. An asterisk indicates a statistically significant difference between FG and SG groups (*p* < 0.05).

The relative abundance profiles of key bacterial taxa showed clear divergences between FG and SG eels. *B*. *tropicus* was markedly enriched in FG individuals, representing a dominant component of their gut microbiota (Figure 6A). In contrast, *Aeromonas jandaei* displayed significantly higher abundance in SG eels (Figure 6B). Likewise, *Edwardsiella tarda* was substantially elevated in SG eels while remaining nearly absent in the FG group (Figure 6C). All three taxa exhibited statistically significant differences between groups (*p* < 0.05), demonstrating distinct microbial signatures that correspond to the contrasting growth phenotypes of the eels.

### 3.2. Selection of Potential Probiotic Based on the Activity of Digestive Enzyme

A total of eight bacterial isolates, which were more abundant in FG samples compared to SG samples, were identified in this study. These isolates were designated as FG1 through FG8. All strains were rod-shaped, motile, and Gram-positive, displaying similar colony morphology characterized by off-white, circular colonies.

To identify potential probiotic strains associated with growth promotion, isolates with higher digestive enzyme activities were selected for further analysis. Protease activity was initially assessed among the isolates. The results revealed that strain FG2 exhibited the highest protease activity, which was significantly greater than that of strains FG3, FG5, and FG6. However, no significant differences in protease activity were observed among strains FG1, FG2, FG4, FG7, and FG8 (Table 4). Based on these findings, strains FG1, FG2, FG4, FG7, and FG8 were further evaluated for additional digestive enzyme activities.

Table 5 presents the comparative analysis of digestive enzyme activities, including lipase, amylase, xylanase, cellulase, and phytase, among the selected isolates. Strain FG2 demonstrated the highest activities for lipase, amylase, cellulase, and phytase, outperforming the other strains in most cases. For lipase activity, strain FG2 did not show a significant difference compared to strains FG4 and FG8 but exhibited significantly higher activity than strains FG1 and FG7. In terms of amylase activity, no significant differences were detected among strains FG1, FG2, and FG7, whereas strains FG4 and FG8 demonstrated significantly lower amylase activity compared to strain FG2. In contrast to other enzymes, the highest xylanase activity was observed in strain FG7, which was significantly greater than the activities detected in strains FG1, FG2, FG4, and FG8. For cellulase activity, no significant differences were observed among strains FG2, FG4, and FG7, although strains FG1 and FG8 displayed significantly lower cellulase activity compared to strain FG2. Notably, the highest phytase activity was also recorded in strain FG2, which was significantly greater than that of all other strains.

### 3.3. Bacterial Identification

**Figure 7:**
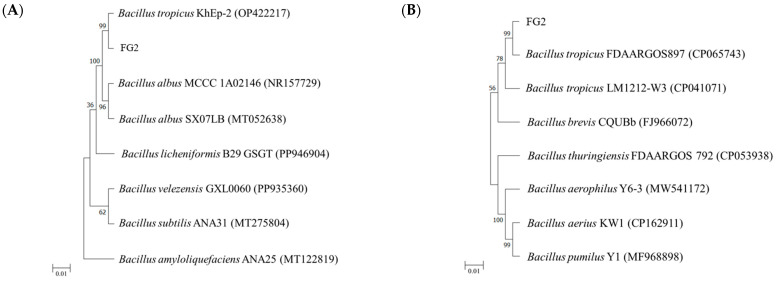
Phylogenetic tree constructed using the Neighbor-Joining method, based on 16S rDNA (**A**) and *gyrB* (**B**) sequences from strain FG2 and closely related taxa within the *Bacillus tropicus*. The scale bar represents the phylogenetic distance, corresponding to 0.01 nucleotide substitutions per site.

The 16S rDNA and *gyrB* sequences were utilized to identify the FG2 isolate, which exhibited the highest digestive enzyme activities as compared to other isolates. A 1441 bp 16S rDNA sequence was determined and subsequently deposited in the GenBank database under the accession number PQ895646. To elucidate the phylogenetic relationships between the FG2 isolate and known isolates, a combined dataset of 16S rDNA sequences for *Bacillus* species was retrieved from the NCBI database and used to construct a phylogenetic tree (Figure 7A). The analysis revealed that the FG2 isolate shared 100% sequence similarity with *B. tropicus* (GenBank accession number: PQ895646) and *B. albus* (GenBank accession numbers: NR157729 and MT052638).

To further confirm the species designation of the FG2 isolate, *gyrB* sequencing was performed. A 970 bp *gyrB* sequence was determined and subsequently deposited in the GenBank database under the accession number PV016491. The *gyrB* sequence analysis demonstrated that FG2 exhibited 98.04% to 99.79% sequence similarity to *B. tropicus* (GenBank accession numbers: CP065743 and CP041071). Phylogenetic analysis grouped FG2 within the same clade as *B. tropicus*, supported by a strong bootstrap value (Figure 7B). Based on the morphological characteristics, 16S rDNA sequence analysis, *gyrB* sequence analysis, and phylogenetic relationships, the FG2 isolate was conclusively identified as *B. tropicus*.

### 3.4. Virulence Assessment

To evaluate the virulence of *B. tropicus* FG2, a virulence assessment was conducted on a range of aquaculture species, including *A. bicolor pacifica*, *Oreochromis* sp., *E. fuscoguttatus*♀ × *E. lanceolatus*♂, *P. vannamei*, *P. monodon*, and *M. rosenbergii*. The injection method was employed to administer *B. tropicus* FG2 to the test organisms. Over the course of the 14-day experimental period, no mortality or clinical abnormalities were observed in any of the tested fish and crustacean (Table 1). These findings confirm the non-virulent nature of *B. tropicus* FG2 and support its potential application as a probiotic in aquaculture.

### 3.5. Growth Performance of Eels Fed with Probiotic

The growth performance of eels fed with the control diet and the diet supplemented with the probiotic *B. tropicus* FG2 at a concentration of 10^9^ cfu (kg diet)^−1^ is presented in Table 6. The inclusion of the dietary probiotic significantly enhanced growth performance, as evidenced by increased final weight, percentage weight gain, and total production per tank in the probiotic-treated group compared to the control group. In contrast, no significant differences were observed between the groups in terms of survival rate, feed efficiency, and condition factor.

### 3.6. Proximate Composition of Dorsal Muscle

The effects of dietary probiotic supplementation on the proximate composition of eel dorsal muscle are presented in Table 7. No significant differences were observed in the moisture, crude protein, or ash content of the dorsal muscle between eels fed the control diet and those fed the probiotic-supplemented diet for 56 days. However, the lipid content of the dorsal muscle was significantly higher in eels fed the probiotic-supplemented diet compared to the control group.

## 4. Discussion

The intestinal microbiota comprises a diverse and dynamic community of microorganisms that inhabit the gastrointestinal tract of the host. These microorganisms play an essential role in a wide array of biochemical processes, including nutrient digestion and absorption, metabolic regulation, and the modulation of host growth and development [12,34,35]. Furthermore, the intestinal microbiota is critical for the development and regulation of the host’s immune system, acting as a pivotal mediator of immune responses and contributing to the maintenance of homeostasis [36]. It has been well-documented that the composition of the gut microbiota is influenced by factors such as host age, nutritional status, and environmental conditions [35,37,38].

In recent years, increasing attention has been directed toward understanding the gut microbiota as a key intrinsic factor in host health and growth. The microbial community not only contributes to nutrient metabolism but also plays a vital role in supporting host growth and mitigating disease risks [19,25,36]. For instance, Sun et al. [21] demonstrated that differences in the gut microbiota were evident between slow-growing and fast-growing groupers, *E. coioides*. In their study, four *Vibrio* species were isolated from the gut of slow-growing groupers, whereas only two *Vibrio* species were detected in fast-growing groupers. Additionally, potential probiotic species such as *B. pumilus*, *B. clausii*, and *Psychrobacter* sp. were found to dominate the gut microbiota of fast-growing groupers. Similarly, variations in gut microbial composition have been observed in eels with different growth rates. In European eels, Fusobacteria were the dominant phylum in fast-growing individuals, while Spirochaetes prevailed in slow-growing counterparts. Furthermore, the relative abundances of *Plesiomonas*, *Turicibacter*, *Nitrospira*, and *Lachnospiraceae bacterium NK4A136* were positively correlated with growth rates [22]. In swamp eels, *Monopterus albus*, notable differences in bacterial composition were also recorded between small and large individuals. Burkholderiales were significantly more abundant in swamp eels, whereas Clostridiales exhibited higher proportions in larger ones. Additionally, almost all Fusobacteria detected in the gut of larger eels belonged to the genus *Cetobacterium* [39]. In American eels, notable shifts in the relative abundances of bacterial phyla were observed between slow-growing and fast-growing individuals. Slow-growing eels exhibited higher relative abundances of Proteobacteria (74.17%) compared to fast-growing eels (59.08%), whereas Firmicutes and Bacteroidota were enriched in fast-growing eels. At the genus level, potential pathogenic bacteria, including *Pseudomonas* and *Mycoplasma*, were predominant in slow-growing eels. In contrast, several genera within the Firmicutes phylum (*Coprococcus*, *Dialister*, *Clostridium*, *Dorea*, and *Blautia*) and the genus *Larkinella* within the Bacteroidota phylum were significantly enriched in fast-growing eels [12]. Many of these genera, identified in fast-growing American eels, are considered potential probiotics [40,41]. In the present study, similar patterns of microbial variation were observed between fast-growing and slow-growing short-finned eels. The dominant phyla included Firmicutes, Proteobacteria, Bacteroidota, and Fusobacteria. Consistent with findings in American eels [12], fast-growing short-finned eels exhibited higher relative abundances of Firmicutes and Bacteroidota, while Proteobacteria were more abundant in slow-growing individuals. At the species level, potential pathogenic bacteria such as *E. tarda* and *A. jandaei* were more prevalent in slow-growing eels, whereas the probiotic bacterium *B. tropicus* was enriched in fast-growing eels. The higher abundance of beneficial bacteria in fast-growing eels, coupled with the reduced presence of pathogenic bacteria, likely contributes to enhanced growth performance and a lower risk of disease, as observed in both the current study and prior research on American eels [12].

The relative abundance of bacteria, including *E. tarda* and *A. jandae*, identified in slow-growing eels, has been recognized as significant pathogens in aquaculture fish [42,43]. Among them, *E. tarda* is a primary etiological agent of Edwardsiellosis, a severe systemic bacterial disease that affects various fish species, including eels [42,44]. Alcaide et al. [44] reported the isolation of *E. tarda* from body ulcers and internal organs of wild European eels in a Mediterranean freshwater coastal lagoon. All isolates of *E. tarda* demonstrated virulence in eels, with lethal dose 50% (LD50) values ranging from 10^4.85^ to 10^6.83^ cfu per individual, confirming their role as the causative agents of the hemorrhagic disease observed in wild European eels. Edwardsiellosis caused by *E. tarda* has also been diagnosed in farmed *A. marmorata*, leading to high mortality rates. Affected eels exhibited severe hemorrhages in multiple organs, increased melano-macrophage activity in the spleen and kidney, and cytoplasmic vacuolation along with liquefactive necrosis in the brain [45]. Similarly, outbreaks of Edwardsiellosis caused by *E. tarda* have been reported in farmed *A. japonica*, resulting in substantial mortality [42]. Therefore, the higher abundance of *E. tarda* observed in the intestines of slow-growing eels suggests that these eels are not only characterized by reduced growth rates but are also at an elevated risk of developing Edwardsiellosis, particularly under suboptimal culture conditions.

In addition to *E. tarda*, *Aeromonas* has been identified as another significant pathogen affecting cultured eels. For instance, *A. hydrophila*, a common bacterium in freshwater fish, has been demonstrated to be a fully pathogenic agent in eels [46]. This Gram-negative pathogen, characterized by its short rod-shaped morphology, motility, fermentative metabolism, and β-hemolytic activity, was isolated from diseased short-finned eels exhibiting abnormal behavior and experiencing high mortality rates [47]. Both *A. hydrophila* and *A. jandaei* have been confirmed as pathogenic bacteria in European eels, with O-serotypes serving as critical epidemiological markers for motile *Aeromonas* strains pathogenic to eels [48]. *A. jandaei* is a mesophilic species typically associated with opportunistic infections in fish [48,49]. Strains of *A. jandaei* have frequently been isolated from cultured eels suffering from “red fin disease” [50,51]. In this study, *A. jandaei* was found to be relatively more abundant in the gut of slow-growing eels compared to fast-growing eels. This observation suggests that slow-growing eels may be more susceptible to developing red fin disease, particularly under deteriorating environmental conditions.

*B. tropicus* is a Gram-positive, rod-shaped bacterium characterized by parallel-sided cells with predominantly bluntly rounded ends, though truncation may occasionally be observed. This microorganism exhibits morphological plasticity, appearing as single cells, pairs, unjointed filaments, and both short and long chains. Sporulation occurs rapidly on solid media, typically within 24 h; however, in liquid media, spore formation is comparatively delayed. Notably, spores are not detected in vivo during the organism’s lifecycle. *B. tropicus* is motile, possessing robust peritrichous flagella. Its colony morphology closely resembles that of *B. anthracis* [52], albeit with less pronounced curling at the colony margins. In certain cases, the colonies exhibit a perforated appearance, whereas in its shorter form, they appear granular with irregular borders [52]. Functionally, *B. tropicus* is recognized as a beneficial bacterium, particularly due to its production of keratinase, an enzyme capable of degrading poultry feathers [53]. Additionally, *B. tropicus* has been isolated from the intestinal tract of tilapia and identified as a potential probiotic, given its antagonistic activity against multiple aquaculture pathogens, including *Staphylococcus aureus*, *Pseudomonas aeruginosa*, and *S. pyogenes* [53]. In the present study, the relatively higher abundance of *B. tropicus* in fast-growing eels may be linked to a concomitant reduction in the abundance of pathogenic bacteria within the intestinal microbiota, suggesting a potential role in promoting host health.

In this study, *B. tropicus* FG2, a strain associated with rapid growth, was successfully isolated from the intestines of fast-growing eels. This strain was selected based on its robust production of digestion-related enzymes, including protease, lipase, amylase, xylanase, cellulase, and phytase. The exceptional enzymatic profile of *B. tropicus* FG2 highlights its potential as a probiotic candidate in aquaculture systems. A primary benefit of probiotics in aquaculture is their ability to enhance nutrient utilization in host. This is achieved through the production of supplemental digestive enzymes and the degradation of anti-nutritional factors in feed. Probiotic-derived enzymes maintain functionality as the probiotics pass through the host’s gastrointestinal tract, eventually colonizing and proliferating in the intestines. Proteases, for instance, catalyze the hydrolysis of proteins into peptides and free amino acids, which are readily absorbed by intestinal epithelial cells. Numerous studies have demonstrated the growth-promoting effects of protease-producing probiotics in aquaculture species, such as white shrimp, *P. vannamei* [54] (Liu et al., 2009) and parrotfish, *Oplegnathus fasciatus* [55]. In addition to protease, *B. tropicus* FG2 exhibits the ability to produce amylase, cellulase, and xylanase. These enzymes are essential for the digestion of carbohydrates, such as starch, cellulose, and hemicellulose, which are abundant in aquafeeds. For example, *B. safensis* NPUST1, another probiotic strain capable of producing these enzymes, was incorporated into the diet of Nile tilapia, *Oreochromis niloticus*, resulting in significant improvements in growth performance and feed efficiency [56]. Phytic acid, a common anti-nutrient found in plant-based feed ingredients, chelates essential minerals such as iron, zinc, calcium, and phosphate, thereby reducing their bioavailability in the host’s intestines [57]. The enzyme phytase hydrolyzes phytic acid, liberating these bound minerals and improving their absorption. This has been demonstrated in *B. subtilis* E20, whose phytase activity effectively degrades phytic acid, leading to enhanced nutrient utilization and growth in aquatic animals [58]. Given the enzymatic capabilities of *B. tropicus* FG2, including its production of protease, lipase, amylase, cellulase, xylanase, and phytase, it is reasonable to infer that this strain contributes to the digestion and absorption of proteins, lipids, carbohydrates, and minerals in aquaculture species. These mechanisms likely underlie the observed improvements in feed efficiency and growth performance, thereby highlighting the potential of *B. tropicus* FG2 as a probiotic for application in aquaculture.

*Bacillus* species have been extensively utilized as probiotics to enhance the growth performance and health of aquaculture species [59]. *Bacillus* is a Gram-positive, rod-shaped, spore-forming bacterium that is either aerobic or facultatively anaerobic, allowing it to thrive in diverse environments. These bacteria can be isolated from various sources, including soil, air, water, animal intestines, vegetables, and fermented foods [60,61]. This ecological versatility underscores their adaptability and potential as probiotics in aquaculture systems. Despite their benefits, certain *Bacillus* species have been identified as opportunistic pathogens or producers of toxins that can pose risks to host animals. Therefore, it is essential to evaluate the safety of specific *Bacillus* strains before their application as probiotics [54]. In the present study, the safety of *B. tropicus* FG2 was rigorously assessed for its application in aquaculture. This evaluation involved high-dose (10^9^ cfu per gram of body weight) injections in six aquatic species, including 3 fish species and 3 crustaceans. No mortalities were observed in any of the tested animals, indicating that *B. tropicus* FG2 does not exhibit acute pathogenicity or toxicity under the conditions tested. These findings suggest that *B. tropicus* FG2 is a safe candidate for use as a probiotic in aquaculture.

Beyond safety, *B. tropicus* FG2 has shown the potential to enhance the growth performance of short-finned eels when incorporated into their diet at a concentration of 10^9^ cfu per kg of feed. A subsequent investigation, consistent with the present study, also demonstrated that dietary supplementation with *B. tropicus* FG2 significantly enhanced the growth performance of short-finned eels [62]. The observed improvement in growth performance may be attributed to the digestive enzymes produced by the probiotic, which enhance nutrient utilization. In addition to its impact on growth performance, dietary supplementation with *B. tropicus* FG2 resulted in an increase in lipid content in the dorsal muscle of short-finned eels. This effect may be associated with the higher lipase activity of *B. tropicus* FG2, which likely enhances lipid digestion and absorption, leading to greater lipid deposition in muscle tissues.

Although the current findings highlight the dual benefits of *B. tropicus* FG2 in promoting both growth and nutritional composition, certain limitations should be acknowledged. In this study, the intestinal microbiota composition of eels was not analyzed following probiotic feeding, and the persistence or colonization capacity of *B. tropicus* FG2 in the gut was not directly determined. Given that *B. tropicus* FG2 was originally isolated from the eel intestine, its potential for colonization is plausible; however, direct evidence remains lacking. Future studies should therefore integrate high-resolution microbiome profiling (e.g., 16S rRNA sequencing, metagenomics) and strain-specific tracking approaches (e.g., qPCR, genome-based markers) to elucidate how *B. tropicus* FG2 modulates gut microbial ecology and contributes to digestive physiology and host health. Such mechanistic insights, together with long-term assessment and on-farm validation under commercial rearing conditions, are essential to fully establish the applicability and functional stability of *B. tropicus* FG2 as a next-generation probiotic for aquaculture.

## 5. Conclusions

This study provides a comprehensive analysis of the intestinal microbiota of eels, highlighting the taxonomic diversity of bacterial communities in two growth groups: FG and SG. The microbiota of FG eels was characterized by the presence of abundance species, such as *B. tropicus*. In contrast, SG eels harbored the relative abundance of potentially pathogenic bacteria, including *E. tarda* and *A. jandaei*. The isolation of *B. tropicus* FG2 from the intestine of FG eels, selected as a probiotic candidate, was supported by its demonstrated superior digestive enzyme activities. The non-virulent nature of *B. tropicus* FG2, confirmed across multiple aquaculture species, further supports its safety for application in aquaculture. Dietary supplementation of *B. tropicus* FG2 significantly improved growth performance of eels at 10^9^ cfu (kg diet)^−1^. Furthermore, the probiotic-treated group demonstrated an enhanced lipid content in the dorsal muscle without adverse effects on the survival rate. Collectively, these findings underscore the potential of *B. tropicus* FG2 as a probiotic to promote growth of aquaculture eels, offering a sustainable approach to improve production efficiency.

## Figures and Tables

**Table 1:** Virulence assessment and culture environment of aquatic animals after a 14-day challenge with *Bacillus tropicus* FG2 through injection at a concentration of 10^9^ cfu (g weight)^−1^. F: Freshwater; S: Saltwater (35 psu).

Categories	Common Name	Organism	Culture Condition	Body Weight (g)	Mortality (%)
Fish	Short-finned eel	*Anguilla bicolor pacifica*	F	3.29 ± 0.13	0
	Tilapia	*Oreochromis* sp.	F	3.11 ± 0.08	0
	Hybrid grouper	*Epinephelus fuscoguttatus* ♀ × *E. lanceolatus* ♂	S	51.22 ± 4.27	0
Crustaceans	White shrimp	*Penaeus vannamei*	S	8.37 ± 0.16	0
	Tiger shrimp	*Penaeus monodon*	S	5.62 ± 0.23	0
	Giant freshwater prawn	*Macrobrachium rosenbergii*	F	0.67 ± 0.02	0

**Table 2:** Ingredients of experimental diets.

	Experimental Diets (% on Dry Matter Basis)	
	Control	10^9^
Fish meal	63	63
Soybean meal	4.9	4.9
Alph-starch	19	19
Squid meal	3	3
Chicken meal	3	3
Fish oil	4	4
Vit. premix	1	1
Min. premix	2	2
Skim milk	0.1	0
Probiotic	0	0.1
Proximate composition (%)		
Crude protein	48.2 ± 1.2	48.3 ± 0.9
Crude lipid	9.89 ± 0.8	9.96 ± 0.9
Ash	4.8 ± 0.6	5.1 ± 1.32
Moisture	7.6 ± 1.1	7.3 ± 1.1

**Sources of ingredients:** Fish meal (Trident Seafoods Corporation, Seattle, WA, USA); Soybean meal (a kind gift from Central Union Oil Corp., Taichung, Taiwan); Alph-starch (Ping Tung Foods Corp., Pingtung, Taiwan); Skim milk (Fonterra Brands, Taipei, Taiwan); Squid meal (Lee-Yih Forage Co., Ltd., Pingtung, Taiwan); Fish oil (Der Herng Industrial Co., Ltd., Pingtung, Taiwan); Chicken meal (Sanhe Feed Co., Ltd., Pingtung, Taiwan); Mineral premix and vitamin premix were provided by Nice Garden Industrial Co., Ltd., Taipei, Taiwan.

**Table 3:** Gut microbiome α-diversity between FG and SG eels.

Treatments	Genus	Margalef’s Species Richness (d)	Shannon Index	Simpson Index
FG	121	10.42 ± 2.98	1.69 ± 0.27	0.59 ± 0.10
SG	116	9.67 ± 0.49	2.06 ± 0.18	0.73 ± 0.06
*p*-value		0.777	0.235	0.197

**Table 4:** Protease activity of isolates from the gut of FG eels. Data are expressed as mean ± standard error (S.E.), with different letters indicating significant differences among strains (*p* < 0.05).

Strains	Protease Activity (U mg^−1^)
FG1	849.1 ± 28.6 ^ab^
FG2	867.8 ± 19.9 ^a^
FG3	589.8 ± 14.4 ^d^
FG4	855.4 ± 9.4 ^ab^
FG5	693.3 ± 14.0 ^c^
FG6	798.9 ± 30.6 ^b^
FG7	829.4 ± 12.9 ^ab^
FG8	851.0 ± 32.9 ^ab^
Pr > F	<0.001

**Table 5:** Digestive enzyme activity of isolates from the gut of FG eels. Data are presented as mean ± standard error (S.E.), with different letters indicating significant differences among strains for the same enzyme (*p* < 0.05).

Strains	Lipase (U mg^−1^)	Amylase (U mg^−1^)	Xylanase (U mg^−1^)	Cellulase (U mg^−1^)	Phytase (U mg^−1^)
FG1	17.93 ± 1.79 ^b^	479.89 ± 28.89 ^a^	14.82 ± 1.34 ^c^	98.56 ± 6.20 ^b^	10.88 ± 0.66 ^b^
FG 2	21.45 ± 0.66 ^a^	518.28 ± 47.64 ^a^	24.65 ± 0.88 ^b^	156.14 ± 23.66 ^a^	12.90 ± 0.25 ^a^
FG 4	19.50 ± 1.59 ^ab^	64.86 ± 16.01 ^c^	23.55 ± 0.19 ^b^	123.84 ± 11.18 ^ab^	11.46 ± 0.21 ^b^
FG 7	17.13 ± 0.12 ^b^	368.41 ± 187.38 ^ab^	31.47 ± 2.22 ^a^	155.95 ± 7.30 ^a^	11.39 ± 0.55 ^b^
FG 8	21.36 ± 0.41 ^a^	134.11 ± 76.19 ^bc^	15.52 ± 1.01 ^c^	105.30 ± 3.76 ^b^	11.23 ± 0.14 ^b^
Pr > F	0.028	0.006	<0.001	0.006	0.016

**Table 6:** Growth performance of eels fed a control diet and a diet supplemented with probiotic (10^9^ cfu (kg diet)^−1^) for 56 days. Data are presented as mean ± standard error (S.E.), with asterisks (*) indicating significant differences between treatments (*p* < 0.05).

	Experimental Diets		
	Control	Probiotic	*p*-Value
Final weight (g)	26.9 ± 0.9	30.4 ± 0.7 *	0.048
Survival (%)	99.3 ± 0.8	98.8 ± 1.2	0.124
Percentage of weight gain (%)	162.6 ± 12.8	196.7 ± 6.7 *	0.021
Feed efficiency	0.62 ± 0.04	0.63 ± 0.02	0.258
Total production (g) per tank	2671.5 ± 145.8	2943 ± 37.7 *	0.048
Condition factor	2.16 ± 0.09	2.24 ± 0.14	0.564

**Table 7:** Proximate composition of the dorsal muscle of eels fed a control diet and a diet supplemented with probiotic (10^9^ cfu (kg diet)^−1^) for 56 days. Data are presented as mean ± standard error (S.E.), with asterisks (*) denoting significant differences between treatments (*p* < 0.05).

Treatments	Moisture	CP	Lipid	Ash
Control	72.2 ± 0.5	16.0 ± 0.4	9.8 ± 0.4	0.8 ± 0.2
Probiotic	71.3 ± 0.9	16.3 ± 0.4	11.1 ± 0.2 *	0.8 ± 0.1
*p*-Value	0.123	0.171	0.006	0.078

## Data Availability

The 16S rRNA and gyrB sequence data generated in this study have been deposited in the NCBI Sequence Read Archive (SRA) under accession numbers PQ895646 and PV016491, respectively. These datasets are accessible at the following links: https://www.ncbi.nlm.nih.gov/nuccore/2890246720 (accessed on 9 October 2025) and https://www.ncbi.nlm.nih.gov/nuccore/PV016491 (accessed on 5 March 2025), respectively. All other data are available from the corresponding authors upon reasonable request.
